# Mechanism of Salutary Effects of Astringinin on Rodent Hepatic Injury following Trauma-Hemorrhage: Akt-Dependent Hemeoxygenase-1 Signaling Pathways

**DOI:** 10.1371/journal.pone.0025907

**Published:** 2011-10-11

**Authors:** Fu-Chao Liu, Tsong-Long Hwang, Ying-Tung Lau, Huang-Ping Yu

**Affiliations:** 1 Department of Anesthesiology, Chang Gung Memorial Hospital, Taoyuan, Taiwan; 2 College of Medicine, Chang Gung University, Taoyuan, Taiwan; 3 Graduate Institute of Natural Products, Chang Gung University, Taoyuan, Taiwan; 4 Department of Physiology and Pharmacology, Chang Gung University, Taoyuan, Taiwan; 5 Department of Cosmetic Science, Chang Gung Institute of Technology, Taoyuan, Taiwan; Rutgers University, United States of America

## Abstract

Astringinin can attenuate organ injury following trauma-hemorrhage, the mechanism remains unknown. Protein kinase B/hemeoxygenase-1 (Akt/HO-1) pathway exerts potent anti-inflammatory effects in various tissues. The aim of this study is to elucidate whether Akt/HO-1 plays any role in astringinin-mediated attenuation of hepatic injury following trauma-hemorrhage. For study this, male Sprague-Dawley rats underwent trauma-hemorrhage (mean blood pressure 35–40 mmHg for 90 min) followed by fluid resuscitation. A single dose of astringinin (0.3 mg/kg body weight) with or without a PI3K inhibitor (wortmannin) or a HO antagonist (chromium-mesoporphyrin) was administered during resuscitation. Various parameters were measured at 24 h post-resuscitation. Results showed that trauma-hemorrhage increased plasma aspartate and alanine aminotransferases (AST and ALT) concentrations and hepatic myeloperoxidase activity, cytokine induced neutrophil chemoattractant (CINC)-1, CINC-3, intercellular adhesion molecule-1, and interleukin-6 levels. These parameters were significantly improved in the astringinin-treated rats subjected to trauma-hemorrhage. Astringinin treatment also increased hepatic Akt activation and HO-1 expression as compared with vehicle-treated trauma-hemorrhaged rats. Co-administration of wortmannin or chromium-mesoporphyrin abolished the astringinin-induced beneficial effects on post-resuscitation pro-inflammatory responses and hepatic injury. These findings collectively suggest that the salutary effects of astringinin administration on attenuation of hepatic injury after trauma-hemorrhage are likely mediated via Akt dependent HO-1 up-regulation.

## Introduction

Trauma-hemorrhage induces in excessive production of pro-inflammatory mediators, such as cytokines and chemokines, which play a significant role in the development of multiple organ dysfunctions [1]. Following trauma-hemorrhage, neutrophil movement and migration are mediated by multiple adhesion molecules and pro-inflammatory mediators [2]–[4]. The intercellular adhesion molecule (ICAM)-1 enhances firm adhesion of neutrophils to the vascular endothelium and is markedly up-regulated after trauma-hemorrhage [2], [5]. Furthermore, there is convincing evidence that interleukin (IL)-6 plays a significant role of organ injuries and is required for expression of adhesion molecules and release of chemokines [2], [6]. Chemokines, such as cytokine-induced neutrophil chemoattractant (CINC)-1 and CINC-3, are potent chemoattractants for neutrophils [2], [5].

The phosphatidylinositol 3-kinase (PI3K)/protein kinase B (PKB; Akt) is known to be an endogenous negative regulatory function, which serves to limit pro-inflammatory mediators and chemotactic events in response to injury [7]–[9]. PI3K/Akt pathway also play a pivotal role in the ability of neutrophils to undergo chemotaxis [10], [11]. Inhibition of the PI3K/Akt pathway with a PI3K inhibitor wortmannin increases neutrophil migration to chemotaxis [12] and increases serum cytokine levels in septic mice [8], [13]. Studies have also shown that activation of the PI3K pathway protects organs or cells against ischemia-reperfusion injury and hypoxia through suppression of the apoptosis machinery [14]. There is now considerable evidence demonstrating an important role of PI3K/Akt in reducing neutrophil infiltration and production of cytokines [15].

A growing body of evidence indicates that Akt activation induces hemeoxygenase (HO)-1 [16], [17], which is known to play a protective role in many organs under various deleterious conditions, including trauma-hemorrhage [18], [19]. Up-regulation of HO-1 causes a reduction of cytokines, adhesion molecules, chemokines, and neutrophil accumulation, and ameliorates organ injury in shock status [20], [21]. Studies have also shown that administration of 17β-estradiol, flutamide, or resveratrol after trauma-hemorrhage increased HO-1 expression, which prevents the organs from dysfunction and injury [18], [19], [21]–[23].

The liver is considered to be a critical organ following trauma-hemorrhage. Hepatic dysfunction reflects the severity of tissue injury and is associated with outcome of survival. Studies have indicated that overproduction of those chemokines leads to hepatic injury after trauma-hemorrhage [2], [15]. Astringinin (piceatannol), a resveratrol analogue with higher antioxidant activity and greater radical scavenging capacity than resveratrol, has been shown to possess anti-arrhythmic, anti-tumorigenic, and apoptosis-inducing effects [24]–[27]. Previous studies have shown that astringinin can reduce cytokine production and demonstrates cardioprotective activities after shock-like states in ischemic-reperfused rat hearts [28]. Our recent study also shown that astringinin can attenuate hepatic injury after trauma-hemorrhage through inhibit of pro-inflammatory mediator production [29]. However, it remains unknown whether Akt/HO-1 play a critical role in the astringinin-mediated organ protection after trauma-hemorrhage. We hypothesized that the beneficial effects of astringinin after trauma-hemorrhage are mediated via an Akt-dependent up-regulation of HO-1. To test this hypothesis, we treated animals with astringinin alone and in combination with the PI3K inhibitor wortmannin or the HO antagonist chromium-mesoporphyrin after trauma-hemorrhage. The effects of these treatments were then examined to plasma alanine aminotransferase (ALT) and aspartate aminotransferase (AST) levels as well as hepatic tissue myeloperoxidase (MPO) activity, IL-6, ICAM-1, chemokine (CINC-1 and CINC-3) levels, and Akt/HO-1 expression after trauma-hemorrhage.

## Materials and Methods

### Animals

Adult male (275–325 g) Sprague-Dawley rats were used in this study. The rats were obtained from the National Science Council and were allowed to acclimatize to the animal facility for 1 wk before the experiments. All animal experiments were performed according to the guidelines of the *Animal Welfare Act* and *The Guide for Care and Use of Laboratory Animals* from the National Institutes of Health and approved by the Institutional Animal Care and Use Committee of Chang Gung Memorial Hospital.

### Trauma-Hemorrhage Procedure

Before initiation of the experiment, Male Sprague- Dawley rats were fasted overnight but allowed free water access. Trauma-hemorrhage and resuscitation was then performed as described previously [2]. In brief, rats were anesthetized by isoflurane inhalation, and a 5-cm midline laparotomy was performed to induce soft tissue trauma. The abdominal wound was then closed in layers, and polyethylene catheters (PE-50; Becton Dickinson & Co., Sparks, MD) were placed in both femoral arteries and the right femoral vein, and the incision sites were then closed. The wounds were bathed with 1% lidocaine (Elkins-Sinn Inc., Cherry Hill, NJ) throughout the operative procedure to reduce postoperative pain. The rats were allowed to awaken, after which they were bled rapidly within 10 minutes to a mean arterial pressure of 35 to 40 mmHg. This level of hypotension was maintained until the animals could no longer maintain a mean arterial pressure of 40 mmHg unless some fluid in the form of Ringer's lactate was administered. This time was defined as maximum bleed-out. After the maximal bleed-out, mean arterial pressure was maintained between 35 to 40 mmHg until 40% of the maximal bleed-out volume was returned in the form of Ringer's lactate solution (about 90 min from the onset of bleeding). The rats were then resuscitated with four times the volume of the shed blood with Ringer's lactate for 60 minutes. Thirty minutes before the end of the resuscitation period, the rats received astringinin (0.3 mg/kg, intravenously) [29], astringinin plus the PI3K inhibitor wortmannin (1 mg/kg, intravenously at the beginning of resuscitation), co-administration of astringinin with the HO inhibitor chromium-mesoporphyrin (2.5 mg/kg, intraperitoneally at the beginning of resuscitation), or an equal volume of the vehicle (about 0.2 mL, 10% DMSO). After resuscitation, the catheters were removed, the vessels ligated, and the skin incisions closed with sutures. Sham-operated animals underwent all operative procedures, but neither hemorrhage nor resuscitation was performed. Vehicle or astringinin was also administered in sham-operated rats after catheters were placed. The animals were humanely killed at 24 h after the end of resuscitation or sham operation. In the experiment under review, there were 8 rats in each group.

### Measurement of Hepatic Injury

At 24 h after trauma-hemorrhage or sham operation, blood samples with heparin were obtained and plasma was separated by centrifugation. Hepatic injury was determined by measuring plasma levels of AST and ALT using a colorimetric analyzer (Dri-Chem 3000; Fuji Photo Film Co., Tokyo, Japan).

### MPO Assay

MPO activity in homogenates of whole liver was determined as described previously [2]. Frozen tissue samples were thawed and suspended in phosphate buffer (pH 6.0) containing 0.5% hexadecyltrimethylammonium bromide (Sigma, St. Louis, MO). The samples were sonicated on ice, centrifuged at 12,000 g for 15 minutes at 4°C, and an aliquot was transferred into phosphate buffer (pH 6.0) containing 0.167 mg/mL o-dianisidine hydrochloride and 0.0005% hydrogen peroxide (Sigma). The change in absorbance at 460 nm was measured spectrophotometrically for 5 minutes. MPO activity was calculated using a standard curve that was generated using human MPO (Sigma), and values were normalized to protein concentration.

### Measurement of CINC-1, CINC-3, ICAM-1, and IL-6 Levels

The liver tissues were homogenized in PBS (1∶10 weight∶volume; pH 7.4) containing protease inhibitors (Complete Protease Inhibitor Cocktail; Boehringer, Mannheim, Germany). The homogenates were centrifuged at 2,000 g for 20 minutes at 4°C and the supernatant was analyzed for the presence of CINC-1, CINC-3, ICAM-1, and IL-6 using ELISA kits (R&D, Minneapolis, MN) according to the manufacturer's instructions and as described previously [2]. An aliquot of the supernatant was used to determined protein concentration by the Bio-Rad DC Protein Assay (Bio-Rad, Hercules, CA).

### Western Blot Assay

Protein aliquots were used to determine protein concentration by the Bio-Rad DC Protein Assay (Bio-Rad, Hercules, CA). Samples were mixed with 4× sample buffer and were electrophoresed on sodium dodecyl sulfate-polyacrylamide gels and transferred electrophoretically onto nitrocellulose paper. The membranes were then incubated with antibodies to total Akt protein (1∶ 5000 dilution; Cell Signaling), phospho (p)-Akt (Ser473) (1∶2000 dilution; Cell Signaling Technology Inc., Beverly, MA), antibodies to HO-1 protein (1∶6000 dilution; Chemicon International, Temecula, CA), or antibodies to GAPDH (1∶15000 dilution; Abcam, Cambridge, MA) overnight at 4°C. The membranes were later incubated with horseradish peroxidase-conjugated goat anti-rabbit antibody or goat anti-mouse antibody for 1.5 h at room temperature. After the final wash, membranes were probed using enhanced chemiluminescence (Amersham, Piscataway, NJ) and autoradiographed.

### Statistical Analysis

For statistical analysis we used the InStat 3.0 biostatistics program (Graph Pad Software Inc., San Diego, CA). Results are presented as mean ± standard error of the mean (SEM). The data were analyzed using one way analysis of variance and the Tukey test, and differences were considered significant at *p*<0.05.

## Results

### Alteration in Plasma AST and ALT Levels

**Figure 1:**
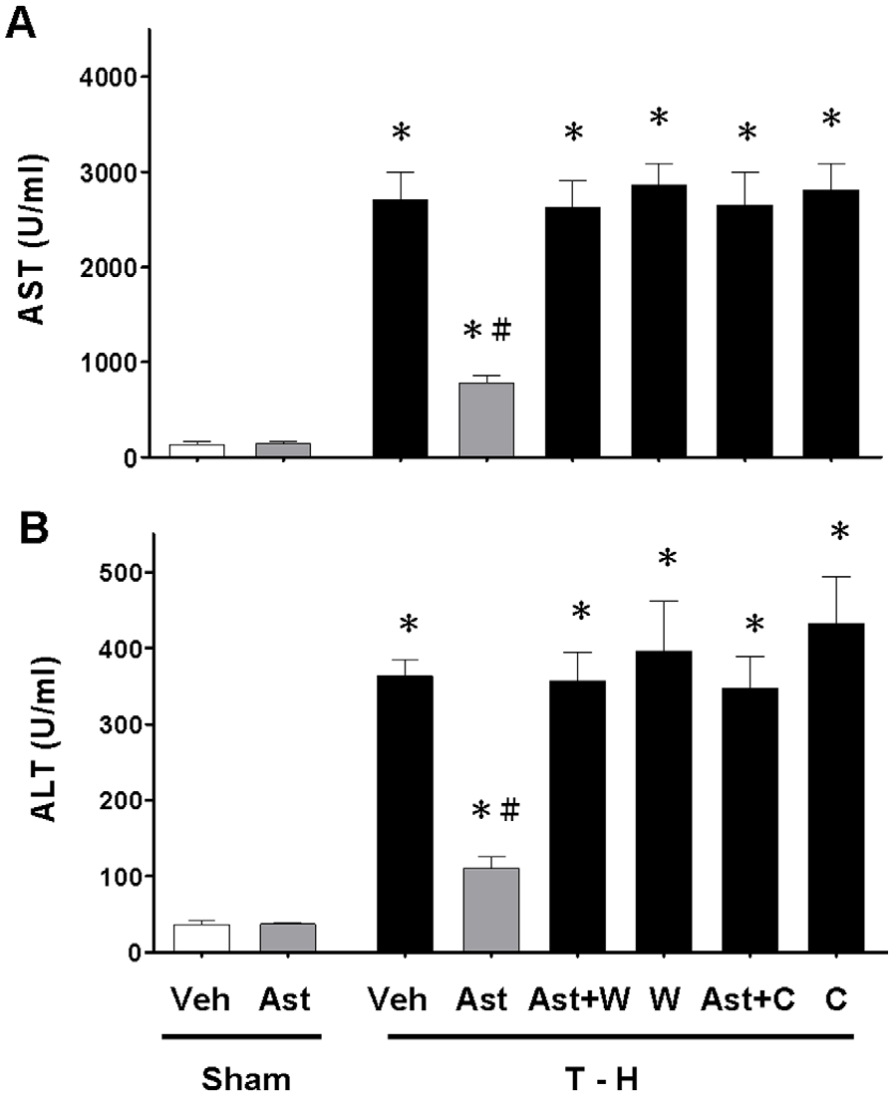
Effect of astringinin treatment on plasma AST (A) and ALT (B) in rats after sham operation (Sham) or trauma-hemorrhage and resuscitation (T-H). Animals were treated with vehicle (Veh), astringinin (Ast), astringinin in combination with wortmannin (Ast+W), wortmannin (W), astringinin in combination with chromium-mesoporphyrin (Ast+C), or chromium-mesoporphyrin (C). Data are shown as mean ± SEM; n = 8 rats in each group. ^*^ *p*<0.05 compared with sham; ^#^ *p*<0.05 compared with T-H+Veh, T-H+Ast+W, T-H+W, T-H+Ast+C, and T-H+C.

As shown in Figures 1A and 1B, no significant difference in plasma AST and ALT levels was observed between vehicle- and astringinin-treated sham groups. At 24 h after trauma-hemorrhage, there were significant increases in plasma AST and ALT levels. Astringinin treatment attenuated the trauma-hemorrhage-induced increase in plasma AST and ALT levels. To determine whether the salutary effects of astringinin in attenuating hepatic injury after trauma-hemorrhage were mediated via an Akt-mediated activity, a group of astringinin-treated trauma-hemorrhage rats were administrated with the PI3 K inhibitor wortmannin. The results indicated that administration of the PI3K inhibitor wortmannin prevented the astringinin-induced decrease in plasma AST and ALT levels. To determine whether astringinin reduced hepatic injury after trauma-hemorrhage via a HO-1-mediated pathway, a group of animals were administrated with the HO enzyme inhibitor chromium-mesoporphyrin along with astringinin. The results indicated that administration of chromium-mesoporphyrin along with astringinin prevented the astringinin-induced decrease in plasma AST and ALT levels.

### Alteration in Hepatic MPO Activity

**Figure 2:**
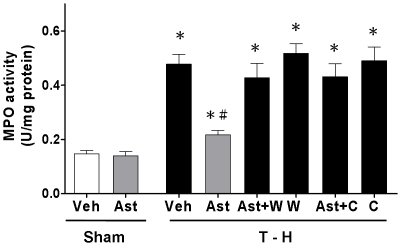
Effect of astringinin treatment on hepatic MPO activity in rats after sham operation (Sham) or trauma-hemorrhage and resuscitation (T-H). Animals were treated with vehicle (Veh), astringinin (Ast), astringinin in combination with wortmannin (Ast+W), wortmannin (W), astringinin in combination with chromium-mesoporphyrin (Ast+C), or chromium-mesoporphyrin (C). Data are shown as mean ± SEM; n = 8 rats in each group. ^*^ *p*<0.05 compared with sham; ^#^ *p*<0.05 compared with T-H+Veh, T-H+Ast+W, T-H+W, T-H+Ast+C, and T-H+C.

The result showed that there were no differences in hepatic MPO activity between vehicle- and astringinin-treated sham groups (Figure 2). After trauma-hemorrhage, MPO activity was increased significantly in vehicle-treated rats compared with sham-operated animals. Astringinin treatment attenuated this increase in hepatic MPO activity. Administration of the PI3K inhibitor wortmannin prevented the astringinin-mediated attenuation of hepatic MPO activity after trauma-hemorrhage. To evaluate the role of HO-1 in the astringinin-induced decrease in hepatic MPO activity in trauma-hemorrhaged rats, rats were treated with the HO inhibitor chromium-mesoporphyrin along with astringinin after trauma-hemorrhage. The results indicated that administration of chromium-mesoporphyrin with astringinin prevented the astringinin-induced decrease in hepatic MPO activity.

### Alteration in Hepatic IL-6 Levels

**Figure 3:**
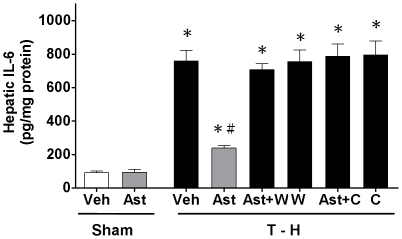
Effect of astringinin treatment on hepatic IL-6 levels in rats after sham operation (Sham) or trauma-hemorrhage and resuscitation (T-H). Animals were treated with vehicle (Veh), astringinin (Ast), astringinin in combination with wortmannin (Ast+W), wortmannin (W), astringinin in combination with chromium-mesoporphyrin (Ast+C), or chromium-mesoporphyrin (C). Data are shown as mean ± SEM; n = 8 rats in each group. ^*^ *p*<0.05 compared with sham; ^#^ *p*<0.05 compared with T-H+Veh, T-H+Ast+W, T-H+W, T-H+Ast+C, and T-H+C.

There was no significant difference in hepatic IL-6 levels between vehicle- and astringinin-treated sham groups (Figure 3). Trauma-hemorrhage significantly increased hepatic IL-6 levels in vehicle-treated rats compared with sham animals, which was attenuated by astringinin treatment. Astringinin-mediated reduction in hepatic IL-6 levels was abolished by wortmannin co-administration. In addition, astringinin-mediated reduction in hepatic IL-6 levels was also abolished by chromium-mesoporphyrin co-administration.

### Alteration in Hepatic CINC-1, CINC-3, and ICAM-1 Expressions

**Figure 4:**
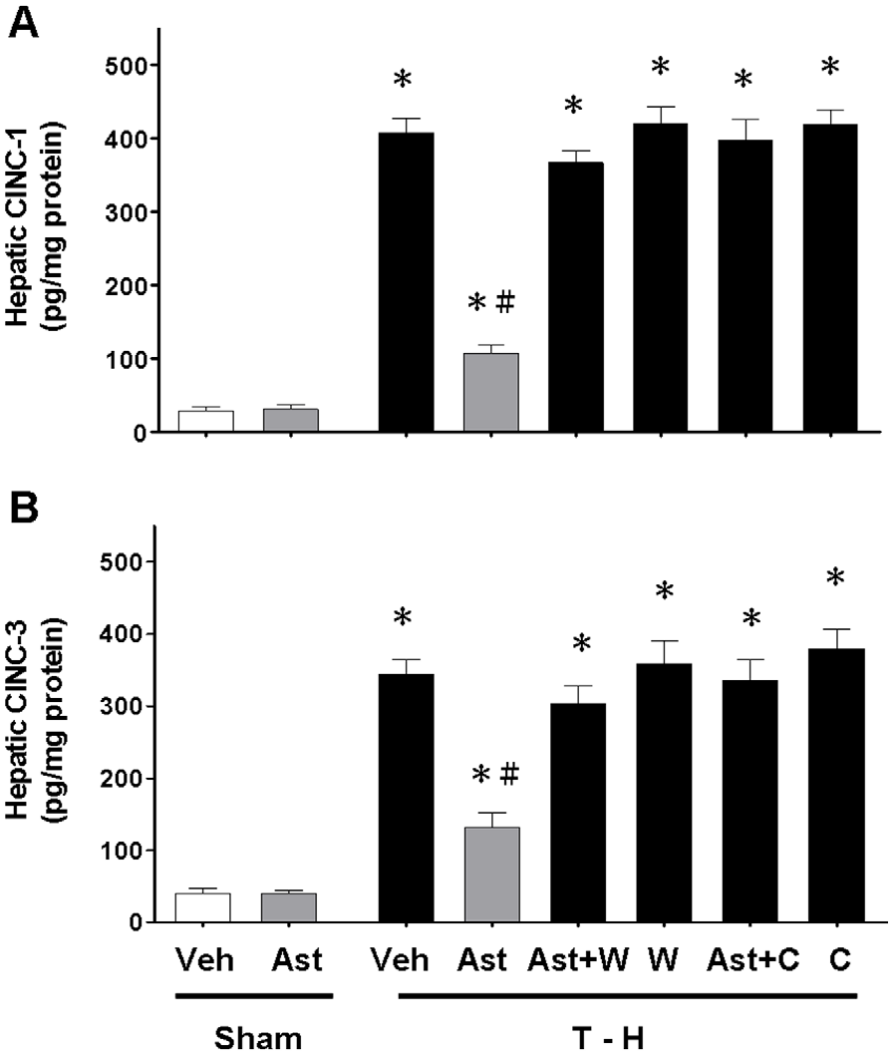
Effect of astringinin treatment on hepatic CINC- 1 (A) and CINC-3 (B) levels in rats after sham operation (Sham) or trauma-hemorrhage and resuscitation (T-H). Animals were treated with vehicle (Veh), astringinin (Ast), astringinin in combination with wortmannin (Ast+W), wortmannin (W), astringinin in combination with chromium-mesoporphyrin (Ast+C), or chromium-mesoporphyrin (C). Data are shown as mean ± SEM; n = 8 rats in each group. ^*^ *p*<0.05 compared with sham; ^#^ *p*<0.05 compared with T-H+Veh, T-H+Ast+W, T-H+W, T-H+Ast+C, and T-H+C.

**Figure 5:**
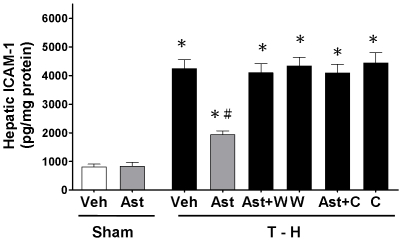
Effect of astringinin treatment on hepatic ICAM-1 levels in rats after sham operation (Sham) or trauma-hemorrhage and resuscitation (T-H). Animals were treated with vehicle (Veh), astringinin (Ast), astringinin in combination with wortmannin (Ast+W), wortmannin (W), astringinin in combination with chromium-mesoporphyrin (Ast+C), or chromium-mesoporphyrin (C). Data are shown as mean ± SEM; n = 8 rats in each group. ^*^ *p*<0.05 compared with sham; ^#^ *p*<0.05 compared with T-H+Veh, T-H+Ast+W, T-H+W, T-H+Ast+C, and T-H+C.

Trauma-hemorrhage significantly increased CINC-1, CINC-3, and ICAM-1 expressions in the liver (Figures 4A and 4B and Figure 5). Treatment with astringinin attenuated trauma-hemorrhage-induced increase in CINC-1, CINC-3, and ICAM-1 expressions. Co-administration of the PI3K inhibitor wortmannin with astringinin prevented the astringinin-induced reduction in CINC-1, CINC-3, and ICAM-1 expressions. Moreover, co-administration of the HO inhibitor chromium-mesoporphyrin with astringinin prevented the astringinin-induced reduction in CINC-1, CINC-3, and ICAM-1 expressions in the trauma-hemorrhaged group.

### Akt Phosphorylation and Protein expression and Activity

**Figure 6:**
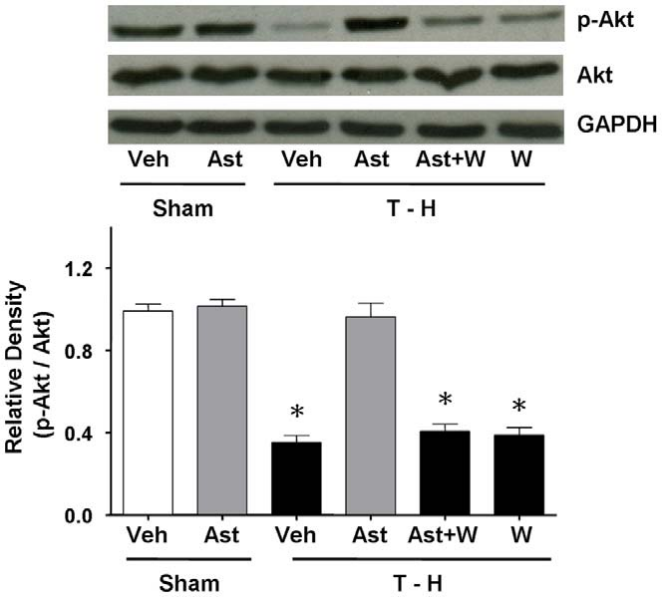
Hepatic p-Akt and Akt protein expressions from shams receiving vehicle (Sham+Veh; lane 1) or astringinin (Sham+Ast; lane 2), trauma-hemorrhage receiving vehicle (T-H+Veh; lane 3), astringinin (T-H+Ast; lane 4), astringinin and wortmannin (T-H+Ast+W; lane 5) or wortmannin (T-H+W; lane 6). Blots were reprobed for GAPDH for equal protein loading in various lanes. The bands were analyzed using densitometry and the values are presented as mean ± SEM; n = 5 rats per group. ^*^ *p*<0.05 versus all other groups.

There was no significant difference in Akt protein expression between sham and trauma-hemorrhaged rats (Figure 6). However, the Akt activity, as determined by its phosphorylation, was significantly decreased after trauma-hemorrhage. Administration of astringinin after trauma-hemorrhage restored Akt activity to the levels observed in sham animals. The increase in p-Akt induced by astringinin was abolished by administration of wortmannin along with astringinin (Figure 6).

### HO-1 Expression in the Liver

**Figure 7:**
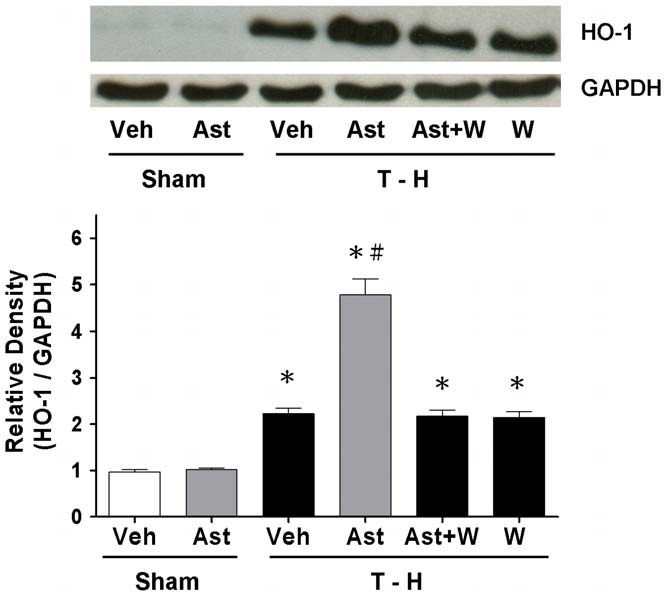
Hepatic HO-1 protein expression from shams receiving vehicle (Sham+Veh; lane 1) or astringinin (Sham+Ast; lane 2), trauma-hemorrhage receiving vehicle (T-H+Veh; lane 3), astringinin (T-H+Ast; lane 4), astringinin and wortmannin (T-H+Ast+W; lane 5), or wortmannin (T-H+W; lane 6). For equal protein loading, membranes were reprobed for GAPDH using mouse monoclonal antibody. The bands were analyzed using densitometry and the values are presented as mean ± SEM; n = 5 rats per group. ^*^ *p*<0.05 compared with sham; ^#^ *p*<0.05 compared with T-H+Veh, T-H+Ast+W, and T-H+W.

Trauma-hemorrhage induced a significant increase in hepatic HO-1 protein expression compared with shams (Figure 7). Administration of astringinin after trauma-hemorrhage induced a further significant increase in hepatic HO-1 protein expression compared with vehicle-treated trauma-hemorrhaged rats. Since astringinin up-regulated hepatic HO-1 after trauma-hemorrhage, we examined whether administration of PI3K inhibitor wortmannin had any effect on HO-1 protein level. The result of these experiments indicated that co-administration of wortmannin prevented the astringinin-induced up-regulation of hepatic HO-1 after trauma-hemorrhage (Figure 7).

## Discussion

The present study indicate that at 24 h after trauma-hemorrhage, plasma ALT and AST concentrations, hepatic MPO activity, IL-6, CINC-1, CINC-3, and ICAM-1 levels are markedly increased in male rats. Administration of a single dose of astringinin during resuscitation could attenuate the increase in those inflammatory markers. Administration of astringinin also prevented the trauma-hemorrhage-induced decrease in hepatic p-Akt expression. A difference in the magnitude of HO-1 expression in the liver of male rats with or without astringinin treatment after trauma-hemorrhage was also found. Administration of the PI3K inhibitor (wortmannin) or the HO inhibitor (chromium-mesoporphyrin) along with astringinin after trauma-hemorrhage abolished the astringinin-induced above effects. These results collectively suggest that the salutary effects of astringinin seem to be mediated via Akt and the up-regulation of HO-1.

Induction of HO-1 plays an important role in organ protection against the deleterious pathophysiological conditions such as trauma-hemorrhage, ischemia, oxidative stress, endotoxemia and wound healing [3], [21], [30], [31]. In this study, we observed that HO-1 level is increased following trauma-hemorrhage and that up-regulation of HO-1 is not entirely dependent on the Akt pathway. This initial increase in HO-1 is likely independent of Akt, as there was a concomitant, significant decrease in Akt phosphorylation in vehicle-treated trauma-hemorrhage rats compared with sham-operated rats. However, the astringinin-mediated increase in HO-1 is found to be Akt-dependent, because co-administration of wortmannin with astringinin abolished the increase in HO-1 and the salutary effects of astringinin treatment in the liver.

Akt phosphorylation is reported to be hepatoprotective after trauma-hemorrhage [15]. Our previous studies have shown that up-regulation of PI3K/Akt pathway attenuates overproduction of cytokines, chemokines, adhesion molecules, and neutrophil accumulation after trauma-hemorrhage [2], [5]. In this study, we found that astringinin-induced attenuation of hepatic injury was likely via increases in Akt activation, which were blocked by the PI3 K inhibitor wortmannin. Our results also suggest that the salutary effects of astringinin are mediated via Akt-dependent HO-1 up-regulation. A growing body of evidence indicates that HO-1 expression is up-regulated after hemorrhagic shock, and that its induction seems to play a central role in the preservation of organ microcirculation under such conditions [21]. The finding that treatment of animals with wortmannin, which blocks Akt, abolished astringinin-induced up-regulation of HO-1 after trauma-hemorrhage suggests that administration of astringinin after trauma-hemorrhage up-regulates HO-1 via the Akt-related pathway.

Chemokines (C-X-C) play an important role in trauma-hemorrhage-induced extravasation of neutrophils into the liver [32]. Previous studies have demonstrated that hepatic injury is associated with an increased neutrophil accumulation [33]. The infiltration of neutrophils in the liver is also accompanied with increased expression of adhesion molecules and elevation of locally produced cytokine/chemokine levels [33]. IL-6 is an important mediator in hepatic inflammation, and is required for adhesion molecule expression and chemokine production [33]. CINC-1 and CINC-3 levels also correlate with tissue MPO activity, a marker of neutrophil infiltration, after trauma-hemorrhage [2]. In this study, our results indicate that trauma-hemorrhage results in a significant increase in hepatic pro-inflammatory cytokine/chemokine levels and ICAM-1 expression, which are accompanied with increased hepatic MPO activity. However, astringinin administration after trauma-hemorrhage attenuated these pro-inflammatory mediators and attenuated hepatic injury under those conditions.

In the present study, astringinin demonstrates a function in regulating the production of pro-inflammatory mediators. The ability of astringinin to modulate expression of inflammatory cytokines as well as chemokines and adhesion molecules suggests a role for astringinin in the regulation of hepatic inflammation. Our study further indicates that astringinin administration after trauma-hemorrhage decreases pro-inflammatory mediator levels and attenuates liver injury likely through Akt-mediated up-regulation of HO-1. Administration of wortmannin or chromium-mesoporphyrin in sham-operated animals was not performed in the present study. Previous studies have shown that administration of P13K inhibitor wortmannin or HO inhibitor chromium-mesoporphyrin does not have additional hepatotoxic effect in the sham-operated rats [18], [34]. It may suggest that P13K inhibitor and HO inhibitor abolish the protective effects of astringinin primarily due to their molecular effect rather than toxic effect itself per se.

In conclusion, this study indicates that astringinin administration ameliorates hepatic injury and IL-6 production after trauma-hemorrhage. The decrease in hepatic injury after astringinin administration is likely due to a reduction of hepatic neutrophil accumulation associated with down-regulation of CINC-1, CINC-3, and ICAM-1. Suppression in hepatic cytokine production by astringinin seems to contribute to the decrease in hepatic expressions of chemokine and adhesion molecule. Blockade of Akt activation or HO-1 and the associated deterioration of the examined parameters suggest that the reduction of neutrophil accumulation in the liver is in part mediated via an Akt dependent HO-1 pathway. Although the precise mechanism of the salutary effects of astringinin in attenuating hepatic injury and the contribution of Akt/HO-1 in protecting against hepatic injury after trauma-hemorrhage remain unclear, our studies provides evidence that Akt-dependent up-regulation of HO-1 may be critical in reducing hepatic injury after trauma-hemorrhage. These findings also have implications for the potential utility of astringinin as a clinical adjunct to trauma-hemorrhage.
